# Hyperbaric Oxygen Therapy Alleviates the Autoimmune Encephalomyelitis via the Reduction of IL-17a and GM-Csf Production of Autoreactive T Cells as Well as Boosting the Immunosuppressive IL-10 in the Central Nervous System Tissue Lesions

**DOI:** 10.3390/biomedicines9080943

**Published:** 2021-08-02

**Authors:** Hsin-Ying Clair Chiou, Shu-Hung Huang, Chih-Hsing Hung, Su-Min Tsai, Hui-Ru Kuo, Yu-Rui Huang, Jiunn-Wei Wang, Szu-Chia Chen, Chao-Hung Kuo, Deng-Chyang Wu, Shau-Ku Huang, Shih-Hsien Hsu, Ming-Hong Lin

**Affiliations:** 1 Center of Teaching and Research, Kaohsiung Municipal Siaogang Hospital, Kaohsiung Medical University, Kaohsiung 812, Taiwan; phoenixchiou@gmail.com; 2 Kaohsiung Medical University Hospital, Kaohsiung Medical University, Kaohsiung 807, Taiwan; 3 Research Center for Environmental Medicine, Kaohsiung Medical University, Kaohsiung 807, Taiwan; pedhung@gmail.com; 4 Department of Surgery, School of Medicine, College of Medicine, Kaohsiung Medical University, Kaohsiung 807, Taiwan; huangsh63@gmail.com; 5 Hyperbaric Oxygen Therapy Center, Kaohsiung Medical University Hospital, Kaohsiung Medical University, Kaohsiung 807, Taiwan; 6 Division of Plastic Surgery, Department of Surgery, Kaohsiung Medical University Hospital, Kaohsiung Medical University, Kaohsiung 807, Taiwan; 7 Department of Pediatrics, School of Medicine, College of Medicine, Kaohsiung Medical University, Kaohsiung 807, Taiwan; 8 Department of Pediatrics, Kaohsiung Municipal Siaogang Hospital, Kaohsiung Medical University, Kaohsiung 812, Taiwan; 9 Department of Microbiology and Immunology, School of Medicine, College of Medicine, Kaohsiung Medical University, Kaohsiung 807, Taiwan; g99231001@gmail.com (S.-M.T.); yrhuang041@gmail.com (Y.-R.H.); 10 Graduate Institute of Medicine, College of Medicine, Kaohsiung Medical University, Kaohsiung 807, Taiwan; a0976566730@gmail.com (H.-R.K.); jackhsu@kmu.edu.tw (S.-H.H.); 11 Department of Internal Medicine, School of Medicine, College of Medicine, Kaohsiung Medical University, Kaohsiung 807, Taiwan; cooljiunn@gmail.com (J.-W.W.); scarchenone@yahoo.com.tw (S.-C.C.); kjh88kmu@gmail.com (C.-H.K.); dechwu@yahoo.com (D.-C.W.); 12 Division of Gastroenterology, Department of Internal Medicine, Kaohsiung Medical University Hospital, Kaohsiung Medical University, Kaohsiung 807, Taiwan; 13 Graduate Institute of Clinical Medicine, College of Medicine, Kaohsiung Medical University, Kaohsiung 807, Taiwan; 14 Department of Internal Medicine, Kaohsiung Municipal Siaogang Hospital, Kaohsiung Medical University, Kaohsiung 812, Taiwan; 15 Division of Nephrology, Department of Internal Medicine, Kaohsiung Medical University Hospital, Kaohsiung Medical University, Kaohsiung 807, Taiwan; 16 National Institute of Environmental Health Sciences, National Health Research Institutes, Miaoli County 350, Taiwan; skhuang@nhri.org.tw; 17 Department of Medicine, Division of Allergy and Clinical Immunology, Johns Hopkins University School of Medicine, Baltimore, MD 21205, USA; 18 Center of Applied Genomics, Kaohsiung Medical University, Kaohsiung 807, Taiwan; 19 Department of Medical Research, Kaohsiung Medical University Hospital, Kaohsiung Medical University, Kaohsiung 807, Taiwan; 20 M.Sc. Program in Tropical Medicine, College of Medicine, Kaohsiung Medical University, Kaohsiung 807, Taiwan

**Keywords:** hyperbaric oxygen, multiple sclerosis, EAE, CNS, Th17, GM-CSF, IL-10

## Abstract

Multiple sclerosis (MS) is a chronic autoimmune disease mainly caused by autoreactive T cells, followed by neuronal demyelination and disabling paralysis. Hyperbaric oxygen therapy (HBOT) is usually an adjunct to therapy for the treatment of neurological disorders. However, it remains still controversial whether HBOT is an effective option for the treatment of MS. Experimental autoimmune encephalomyelitis (EAE) is a well-studied mouse model investigated for the MS pathogenesis and the efficacy of the therapeutic intervention. Both encephalitogenic Th1 and Th17 are pivotal T cell subsets immunopathogenically producing several disease-initiating/modifying cytokines in the central nervous system (CNS) lesions to further exacerbate/ameliorate the progression of EAE or MS. However, it remains unclear whether HBOT modulates the context of T helper cell subsets in CNS lesions. We employed EAE in the presence of HBOT to assess whether disease amelioration is attributed to alterations of CNS-infiltrating T cell subsets. Our results demonstrated that semi-therapeutic HBOT significantly alleviated the progression of EAE, at least, via the suppression of Th17 response, the downregulation of CD4 T helper cells expressing GM-CSF or TNF-α, and the boosting of immunomodulatory IL-4 or IL-10-expressed CD4 T cells in the CNS lesions. Conclusively, HBOT attenuated EAE through the modulation of T cell responses in an earlier stage.

## 1. Introduction

Multiple sclerosis (MS) is a chronic autoimmune and incurable disease of the central nervous system (CNS) manifested by neuronal demyelination and neuro-axonal damage and parenchymal mononuclear infiltration [1]. The etiology of MS is still unclear, potentially being associated with environmental factors and genetic factors. The most common subtype of MS is relapsing-remitting multiple sclerosis (RRMS), in which the clinical symptoms of MS patients usually manifest as relapsing and remitting, in turn, mainly affecting the young adults towards the disabling diseases [2]. The immunopathology of MS is mainly attributed to the dysregulation of disease-initiating autoreactive T cells, producing higher amounts of pro-inflammatory cytokines as well as lower levels of immunosuppressive cytokines [1]. Consequently, the skewed immune deviation is a critical decision repertoire during the pathogenesis of MS. It is getting increasingly evident that multiple immune cell types participate in concert during the initiation and progression of MS [3].

Of note, Hyperbaric oxygen therapy (HBOT) has been reported to treat some neurological disorders, including experimental spinal cord injury [4], brain injury [5], neurodegenerative disease [6], and MS [7,8,9], already for several decades [9,10]. Moreover, it is increasingly evident that HBOT as an adjunct to therapy strikingly promotes the anti-inflammatory responses [11], angiogenesis against hypoxia context [12], and anti-apoptotic capability [13], eventually augmenting the protective roles in neurological disease progression. As reported, MS patients intermittently or consecutively received pure oxygen in the hyperbaric oxygen chamber with an inner pressure of more than 1 atmosphere absolute (ATA), usually 1.75 to 2.5 ATA, for one hour or more once daily over four weeks of treatment. Paradoxically, several clinical trials recommended that HBOT was ineffective for the treatment of MS in previous decades [14,15,16,17,18]. However, there are some reports that HBOT was applied as an adjunct to therapy, and a better remission outcome was observed in MS patients or neuronal cells [7,8,9]. Consequently, the modulatory role of HBOT in MS treatment is still controversial. As known, T cell are an immunopathogenic player in the initiation and progression of MS [1]. However, it remains elusive whether a protective role of HBOT is attributed to T cell modulation and how HBOT modulates the adaptive autoreactive T cells in the CNS lesions of MS patients. To explore whether HBOT modulates the disease-initiating T cell responses of MS, we employed experimental autoimmune encephalomyelitis (EAE) to further decipher the underlying mechanisms.

EAE is a well-established animal model for the investigation of MS and a useful model for the evaluation of the efficacy of therapeutic intervention [19,20]. In the pathogenesis of EAE, mimic the MS in human, both Th1 and Th17 cells, also known as IFN-γ and IL-17A-producing CD4 T helper cells, respectively, are critically immunopathogenic players in the initiation and progression of EAE followed by neuronal demyelination in the CNS, consequently leading to the ascending paralysis of the tail as well as limbs of mice [21,22,23,24]. Impairments of those T helper cell responses contribute to the amelioration of EAE. As is known, Th1 cells produce signature cytokines of IFN-γ and TNF-α to promote CNS inflammation of EAE, however, mice deficient of IFN-γ were not protected from EAE development but limited the progression as well as the severity of the autoimmune disease. On the contrary, Th17 cells secrete IL-17A signature cytokine and mediate an essential role very early in the disease onset of EAE. The deficiency of Th17 cells profoundly ablates the EAE incidence and severity. Moreover, Th17 cells have stunning plasticity, becoming a Th1-like phenotype IFN-γ and IL-17A-double and producing CD4 T cells (ex-Th17) to be a critical player in the pathogenesis of EAE. As investigated, GM-CSF is also produced by both Th1 and Th17 cells, however, it is mainly secreted by Th17cells to be an encephalitogenic cytokine in the CNS lesion of EAE. Meanwhile, GM-CSF is expressed in a novel CD4 T helper subset other than Th1 as well as Th17, being highly associated with the disease severity of EAE [25,26]. Of note, CXCR4 is a chemokine receptor and defines a T helper cell to be highly enriched in the CNS lesion of EAE [26]. Subsequently, CXCR4-expressing CD4 T helper critically modulates the encephalomylitogenicity of the CNS lesions of EAE mice.

However, it is still unclear whether HBOT treatment improves the clinical outcomes of autoimmune encephalomyelitis. To test whether the HBOT is an actual disease-modifying therapy to modulate those CD4 T cell phenotypes, we employed a myelin oligodendrocyte glycoprotein 35–55 (MOG_35–55_)-immunized EAE mouse model in the absence or presence of HBOT, eventually assessing those CD4 population mentioned as above in the CNS lesions of EAE. Our results demonstrated that semi-therapeutic HBOT alleviated the disease progression and clinical severity of MOG-immunized EAE in mice. HBOT suppressed the T cell expansion in the presence of either TCR signaling or autoantigen stimulation. Of note, HBOT decreased the frequency of encephalitogenic Th17 in the CNS lesions of EAE mice and diminished the geometric means of IFN-γ and IL-17A expressions in Th1 and Th17 cells, respectively. Moreover, we found that HBOT markedly dampened the productions of TNF-α and GM-CSF in the CD4 T helper cells in the CNS lesion. On the contrary, we analyzed that HBOT significantly boosted the percentages of immunomodulatory IL-4 and IL-10 of CD4 T helper cells in the CNS of EAE mice, respectively. Conclusively, HBOT modulated the encephalomyelitogencity of T helper cells in the CNS, eventually being highly associated with the attenuation of EAE clinical outcomes.

## 2. Materials and Methods

### 2.1. Animals and Experimental Design

Animals Use Protocol (IACUC-107087) was approved by the Kaohsiung Medical University-Institutional Animal Care and Use Committee. The 8 to 10-week-old female mice were purchased from the National Laboratory Animal Center (Taipei city, Taiwan). Those mice were housed in an Association for Assessment and Accreditation of Laboratory Animal Care International (AAALAC)-accredited facility of Kaohsiung Medical University, which the room temperature of 21 ± 2 °C the humidity of 55 to 70%, and 12:12 h dark/light cycling were stably maintained and controlled in the experimental animal center. After 2 weeks of adaptation, mice were randomly divided into two groups of semi-therapeutic HBOT as well as vehicle control and immunized with MOG antigen to induce autoimmune encephalomyelitis. For exploring the effect of HBOT on EAE clinical manifestations, those immunized mice were followed up for a total of 4 weeks after MOG immunization to determine the EAE disease incidence, day of disease onset, maximal clinical score, and peak phase with the maximal clinical score. For immune cell populations, proliferation assay, and cytokine secretion, the two groups of mice were all sacrificed on day 14 after MOG immunization, and the spleen, spinal cord, and brain were dissected and harvested to prepare the splenic single-cell suspensions and CNS-infiltrating parenchymal mononuclear cells for further analysis [27].

### 2.2. Establishment of EAE and Assessment of Clinical Manifestations

To establish EAE disease animal model, the 10 to 12-week-old C57BL/6J mice were immunized with 100 μg MOG_35–55_ peptides (purity ≥ 98%, MB Co., LTD., Taipei city, Taiwan) emulsified in complete Freund’s adjuvant (CFA) supplemented with 400 mg/mL *Mycobacterium tuberculosis H37Ra* (Difco, Kansas City, MO, USA) on day 0 by the subcutaneous injection into the lateral abdomen [19]. After MOG immunization, those mice were administrated with each shot of 250 ng pertussis toxin (List Biological Laboratories, Campbell City, CA, USA) for a total of two shots on day 0 and 2 by the intraperitoneal injection, subsequently to develop EAE manifested by the paralysis symptoms of tail and limbs. The EAE clinical manifestations were evaluated once per day in the morning by the assignment of scores from 0 to 5 as follows: 0, no clinical sign; 0.5, partial weakness of limb tail; 1, complete paralysis of tail; 2, paralysis of one hind limb; 3, paralysis of both hind limbs; 4, forelimb paralysis; 5, moribund or death [27].

### 2.3. Hyperbaric Oxygen Therapy (HBOT)

To evaluate the therapeutic effects of HBOT in EAE disease, in the semi-therapeutic HBOT group, EAE mice were placed in a hyperbaric oxygen chamber (Genmall Biotech Co. LTD., Taipei city, Taiwan) with pure oxygen once per day for five consecutive days from day 8 to 12 after MOG immunization, followed by a resting of two days. Subsequently, those EAE mice received the second round of HBOT once per day for another five consecutive days from day 15 to 19, for a total of ten times of HBOT for each mouse. The inner pressure of the hyperbaric oxygen chamber gradually raised from 1 to 2.5 ATA over 20 min and then the pressure was maintained at 2.5 ATA for a total of one hour. The aforementioned EAE mice were housed in that chamber with a partition plate to prevent them from closely crowding and they could breathe freely to avoid influencing each other. After the HBOT of one hour, the inner pressure of the chamber was gradually downregulated from 2.5 ATA to normal room oxygen pressure over 20 min. In the vehicle control group, the EAE mice were housed in the cage under the normal room oxygen pressure and moved to the same room of the hyperbaric oxygen chamber [9].

### 2.4. Tissue Preparation

To analyze the underlying molecular mechanisms of HBOT in T cell subsets, the mice were sacrificed by the euthanasia of CO_2_ asphyxiation on day 14 of MOG-immunization for the isolation of CNS-infiltrating parenchymal mononuclear cells for flow cytometric analysis, and the dissection of the spinal cord for histological analysis. For flow cytometric analysis, the CNS-infiltrating parenchymal mononuclear cells were isolated from pooled brain and spinal cord after the transcardiac perfusion of ice-cold PBS buffer as described previously [27]. The CNS tissues were mechanically dissociated through the 100 μm mesh strainer (BD Falcon, San Jose City, CA, USA), and homogenized single-cell suspensions were subsequently fractionated on a 70–30% Percoll gradient by centrifugation at 500× *g* for 30 min at room temperature with the lowest acceleration, at the end of centrifugation without deceleration [27]. The parenchymal mononuclear cells were collected from the interface phase and then washed twice with 1× HBSS (Hank’s Balanced Salt Solution) buffer without phenol red. Consequently, mononuclear cells were counted and then stimulated with 1× Cell Stimulation Cocktail (plus protein transport inhibitors) (eBiosciences, San Diego City, CA, USA) for 4 h at 37 °C in a humidified 5% CO_2_ atmosphere of the incubator. The cocktail-stimulated mononuclear cells were subjected to the procedures of intracellular staining and flow cytometric data were collected by BD LSRII cytometer [27]. For histological analysis, the thoracic spinal cords were dissected after transcardiac ice-cold PBS perfusion, and immediately fixed by 4% formaldehyde (10% formalin) for two days, and embedded in paraffin for tissue slice preparation [27].

### 2.5. Proliferation Assay

To measure the proliferative activity of splenic T cells, the single-cell suspensions were prepared from the spleens of EAE mice on day 14 after MOG immunization. The dissected spleens were homogenized by the glass slides and then filtered with the mesh of 70 μm cell strainer (BD Falcon, San Jose City, CA, USA) for the isolation of splenic single-cell suspensions. For the stimulation with T cell receptor (TCR) signaling of anti-CD3ε monoclonal antibody (mAb) (clone 145-2C11, purchased from BD Pharmingen, San Jose City, CA, USA), splenic single-cell suspensions were seeded as 5 × 10^5^ per well of 96-well plate in the presence of coating 0, 0.3, or 1 μg anti-CD3ε mAb per well, for a total of 48 h. The cell number count of splenic T cells was measured by the CCK-8 colorimetric assay kit. The absorbance of optical density (OD) at 450 nm was measured by a microplate reader and the fold change of absorbance was further calculated by the normalization to the negative control. For the stimulation with MOG_35–55_ antigen, splenic single-cell suspensions, pooled with lymphocytes isolated from inguinal lymph nodes, were seeded as 5 × 10^5^ per well of 96-well plate in the presence of 0, 1, 3 μg/mL MOG_35–55_ per well, for a total of 72 h. The absorbance of OD at 450 nm was used to measure the cell number count of lymphocyte. All data were normalized to negative control and the fold change of the absorbance of OD at 450 nm was calculated and presented as mean ± SEM.

### 2.6. Intracellular Cytokine Staining

To further measure the cytokine expressions of T cells, the CNS-infiltrating parenchymal mononuclear cells were prepared from the EAE mice in the absence or presence of HBOT, as above. For intracellular cytokine staining, those cell suspensions were stimulated with 1× Cell Stimulation Cocktail (plus protein transport inhibitors) (eBiosciences, San Diego, CA, USA), containing phorbol-12-myristate-13-acetate (PMA), ionomycin, brefeldin A and monensin, for 4 h at 37 °C in a humidified 5% CO_2_ atmosphere of the incubator. Surface markers of parenchymal mononuclear cells were stained with fluorochrome-conjugated antibodies for murine CD3ε (145-2C11), CD4 (RM4-5), CD45 (30F11) (purchased from eBiosciences, San Diego City, CA, USA), and CXCR4 (L276F12) (purchased from Biolegend, San Diego, CA, USA) on ice for 30 min. After staining, mononuclear cell suspensions were washed once with 1× FACS (Fluorescence-Activating Cell Sorter) buffer and then fixed with IC fixation buffer (eBiosciences, San Diego, CA, USA) for 20 min at room temperature. Subsequently, those fixed mononuclear cells were washed once with 1× permeabilization buffer (eBiosciences, San Diego, CA, USA) and then were stained with fluorochrome-conjugated antibodies for murine IFN-γ (XMG1.2), TNF-α (MP6-XT22), IL-4 (11B11), IL-10 (JES5-16E3) (purchased from Biolegend, San Diego, CA, USA), IL-17A (TC11-18H10.1), and GM-CSF (MP1-33E9) (purchased from eBiosciences, San Diego, CA, USA) for a total of one hour at room temperature. Subsequently, cell suspensions were washed twice and then suspended in FACS buffer. The flow cytometric data were collected by the BD LSRII cytometer (BD Biosciences, San Jose City, CA, USA). Ultimately, the FlowJo version 10 software was used for data analysis.

### 2.7. ELISA Assay

To measure the productions of cytokines from splenic T cells, the single-cell suspensions were prepared from the spleens of EAE mice. Cells were seeded as 2 × 10^6^ per well of 12-well plate in the presence of coating anti-CD3ε mAb by 0, 0.3, and 1 μg per well for a total of 48 h, respectively. The culture media were collected and immediately stored at −80 °C refrigerator temporarily. The concentrations of IFN-γ and IL-17A in the cell supernatants were measured by the mouse DuoSet ELISA kits according to manufacture instructions (R&D, Minneapolis, MN, USA).

### 2.8. Histological Analysis of Spinal Cord

To analyze the histological context of spinal cord, on day 14 after MOG immunization, the thoracic spinal cords were dissected from EAE mice after transcardiac ice-cold PBS perfusion, and immediately fixed by 4% formaldehyde (10% formalin) for two days and embedded in paraffin for tissue slice preparation. The embedded thoracic spinal cords were cross-sectioned at 5 μm thickness and stained with Hematoxylin and Eosin (H&E) and Luxol Fast Blue (LFB) for identification of CNS-infiltrating parenchymal mononuclear cells as well as the formation of vacuole-like characteristics and myelin integrity, respectively. The tissue sections were examined under a light microscope by the original magnification of 100× or 400× to evaluate demyelination and inflammatory cell infiltration in parenchymal tissues of the spinal cord [27].

### 2.9. Statistical Analysis

The unpaired Student *t*-test and two-way ANOVA test were used to the statistical analysis of all experiments in this study. In a two-way ANOVA test, the unpaired multiple comparisons were subsequently used to calculate the *p* value. * < 0.05, ** < 0.001, *** < 0.0001 was used to distinguish the significance of each comparison.

## 3. Results

### 3.1. Semi-Therapeutic Hbot Ameliorates the Disease Progression and Severity of Autoimmune Encephalomyelitis, as Well as Attenuates the Parenchymal Mononuclear Leukocytes and Demyelination

**Figure 1:**
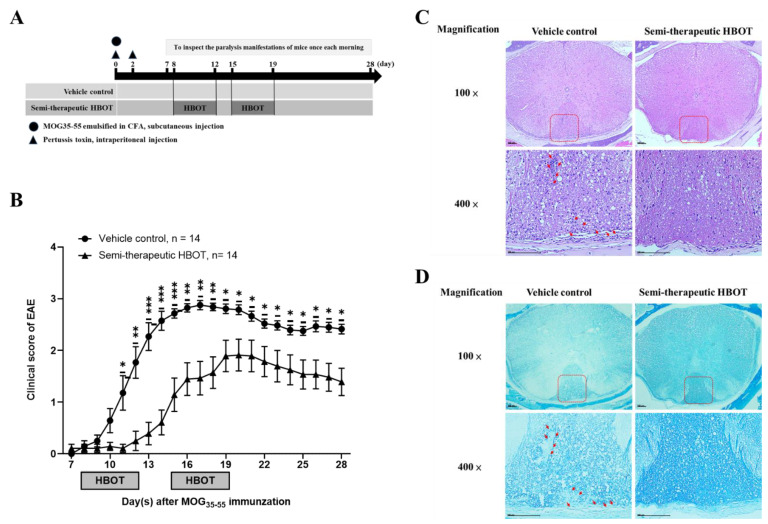
HBOT effectively ameliorated the disease progressions of MOG_35–55_-immunized EAE and improved pathological outcomes of leukocyte infiltration and demyelination. (**A**) Schematic diagram of the procedures of MOG-induced EAE in mice treated with semi-therapeutic HBOT or vehicle control. (**B**) Clinical scores of EAE in mice receiving HBOT (n = 14, filled circle) or vehicle control (n = 14, filled triangle). Clinical manifestations and disease progression of EAE were monitored once in the morning per day from day 7 to 28 after MOG immunization. All data are representative of three independent experiments and were presented as mean ± SEM from fourteen mice in each group. * < 0.05, ** <0.001, or *** < 0.0001 was analyzed by the two-way ANOVA test. (**C**,**D**) Pathological analysis of (**C**) H&E and (**D**) LFB stains of the thoracic spinal cord tissue cross-sections from EAE mice in the absence or presence of HBOT. The thoracic spinal cords were dissected from EAE mice on day 14 after MOG immunization and subsequently were subjected to the procedures of histochemical staining. Representative images are representative of two independent experiments, with at least six mice in each group. The parenchymal mononuclear leukocyte infiltration and vacuolization were displayed as red arrows in the H&E-stained tissue sections (**C**). The signs of neuronal demyelination were indicated as red arrows in the LFB-stained tissue sections (**D**). The original magnifications of images taken by the inverted light microscopy were shown as ×100 and ×400. The scale bars were shown as 100 μm in pictures.

To investigate whether hyperbaric oxygen modulates adaptive immune response, we used EAE animal model in the absence or presence of HBOT to further explore the disease incidence, progression, and clinical manifestation severity. We used MOG_35–55_ antigenic peptides emulsified in the presence of complete CFA supplemented with 4 mg/mL *Mycobacterium tuberculosis* via the subcutaneous route as well as two shots of pertussis toxins via the intraperitoneal route, using all of them to effectively immunize 8-week-old female C57BL/6J mice for establishing EAE [19,20]. After MOG immunization, those mice were placed into the hyperbaric oxygen chamber by one day prior to the average day of disease onset (usually day 9 after MOG/CFA immunization in our laboratory), beginning to manifest tail paralysis-like symptoms, to receive the HBOT as a semi-therapeutic group, or put into a normal room oxygen chamber as a vehicle control group, respectively (Figure 1A). In the semi-therapeutic HBOT group, the MOG-immunized mice received HBOT once per day for five consecutive days (from day 8 to 12) using a hyperbaric oxygen chamber followed by two days for resting without any treatment. Subsequently, those mice received HBOT again once per day for five consecutive days (from day 15 to 19), finally for a total of ten times of treatments per mouse. Mice were housed in the hyperbaric oxygen chamber with the inner pressure of 2.5 ATA for a total of one hour once per day. Our results indicated that, in the vehicle group, C57BL/6J mice were immunized with 100 μg MOG_35–55_/CFA at day 0 followed by two shots of pertussis toxins at day 0 and 2, starting to manifest with partial tail paralysis at day 8 after MOG immunization and reaching the maximal clinical score at day 16 (Figure 1B). However, we found that, in the semi-therapeutic group, HBOT significantly dampened the disease progression and severity of EAE as compared to the vehicle control group, respectively (Figure 1B). The EAE incidence of HBOT-treated mice was slightly lower than that of vehicle control-treated mice (86% v.s. 100%) (Table 1). Besides, HBOT rarely delayed the average numbers of onset days of EAE disease in mice after MOG immunization as compared to those in the vehicle control EAE mice (12.3 ± 1.0 v.s. 10.6 ± 0.5) (Table 1). The maximal clinical scores of EAE mice were strikingly declined in the HBOT semi-therapeutic group as compared to the vehicle control group (2.2 ± 0.3 v.s. 3.1 ± 0.1) (Table 1). Nevertheless, the peak phases in maximal clinical scores rarely distinguished between the semi-therapeutic HBOT and the vehicle control EAE mice (2.4 ± 0.4 v.s. 3.2 ± 0.8) (Table 1). Subsequently, for the examination of histological analysis, tissue cross-sections of the thoracic spinal cord were prepared from the sacrificed EAE mice in the absence or presence of HBOT on day 14 after MOG immunization. By H&E stain, we found that much lower numbers of mononuclear leukocytes in the parenchymal white matter of the thoracic spinal cord from EAE mice treated with HBOT than those from vehicle control-treated ones (Figure 1C). The formation of the vacuole-like bubble was increased in the thoracic spinal cord from vehicle control-treated EAE mice as compared to HBOT-treated mice (Figure 1C). Moreover, by the LFB stain, the demyelination signs of the parenchymal white matter of the thoracic spinal cord were higher in the vehicle control-treated EAE mice than those in the HBOT-treated ones (Figure 1D). Conclusively, our results demonstrated that HBOT attenuated the autoimmune encephalomyelitis to further improve those clinical manifestations of EAE, suggesting the HBOT as an effective strategy against autoimmune diseases.

### 3.2. HBOT Inhibits the Proliferation Activity of T Lymphocytes of MOG-Immunized Mice

**Figure 2:**
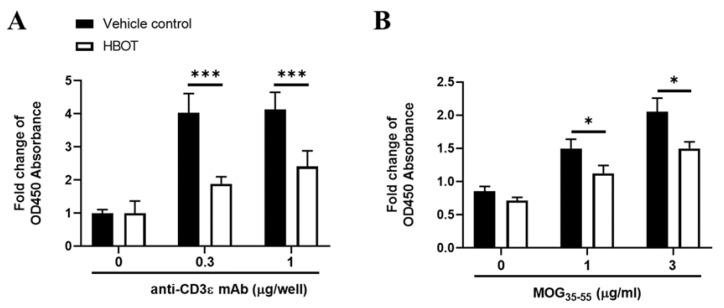
HBOT significantly downregulated the cell expansions of splenic T cells in vitro in the presence of TCR signaling or MOG autoantigen. (**A**,**B**) The proliferation of splenic T cells was measured in the presence of stimulation by TCR signaling (**A**) or autoantigen (**B**), respectively. The single-cell suspensions were isolated from the spleen of EAE mice with (open bar) or without (closed bar) HBOT on day 14 after MOG immunization. Cells were counted then seeded as 5 × 10^5^ per well in a 96-well plate in the presence of the concentrations of 0, 0.3, or 1 μg anti-CD3ε mAb per well for a total culture time of 48 h, respectively. Besides, those isolated single-cell suspensions, pooled with lymphocytes from inguinal lymph nodes, were stimulated with MOG autoantigen by the concentration of 0, 1, or 3 μg/mL MOG_35–55_ peptides for a total stimulation of 72 h, respectively. The cell numbers of cultured splenic T cells were measured by the CCK-8 colorimetric assay kit. The absorbance of OD at 450 nm was measured by a microplate reader and the fold change of absorbance was further calculated by the normalization to the negative control. All data are representative of three independent experiments and were presented as mean ± SEM. The two-way ANOVA test was employed for the statistical analysis. * < 0.05, *** < 0.0001.

To determine whether HBOT influences the immune response of T cells in autoimmune encephalomyelitis, we examined the splenic T cell proliferation of EAE mice in the presence of HBOT manipulation. On day 14 after MOG_35–55_ immunization, HBOT-treated or vehicle control-treated EAE mice were sacrificed, and their spleens were dissected for the isolation of single-cell suspensions. Splenic single-cell suspensions from those mice were stimulated in the presence of TCR signaling for a total of 48 h by plate-coating anti-CD3ε mAb of 0, 0.3, and 1 μg per well, respectively. Our results indicated that the TCR stimulation by both two doses of anti-CD3ε mAbs, the fold changes of cell numbers of splenic T cells were significantly downregulated from HBOT-treated EAE mice as compared to vehicle control-manipulated mice, respectively (Figure 2A). Besides, to test whether HBOT modulates the autoantigen-specific T cell responses, we used splenic single-cell suspensions, pooled with lymphocytes of inguinal lymph node, in the presence of two doses of MOG_35–55_ peptides for the quantification of T cell expansion. We found that the fold changes of T cell expansion in the presence of two different autoantigen doses were markedly diminished in EAE mice with HBOT as compared to those mice treated with normal room oxygen conditions, respectively (Figure 2B). Consequently, these results suggested that HBOT dampened the T cell expansion caused by the stimulation of TCR signaling and autoantigen, respectively, being consistent with the HBOT-mediated amelioration of autoimmune encephalomyelitis in mice.

### 3.3. HBOT Relieves the Inflammatory Responses of Th1 and Th17 in the CNS Lesions of Autoimmune Encephalomyelitis

**Figure 3:**
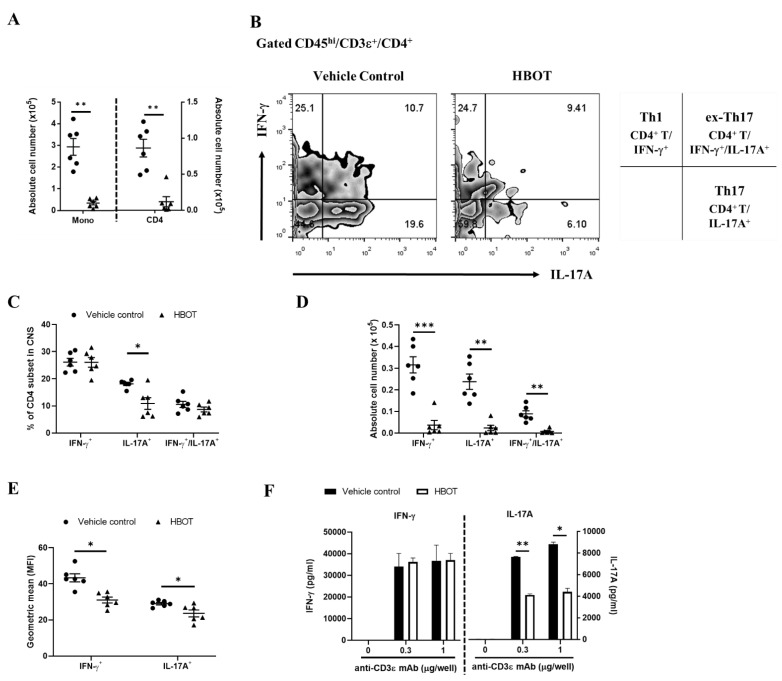
HBOT notably dampened the frequency of the Th17 subset and the signature cytokine expressions of both Th1 and Th17 in the CNS lesions. On day 14 after MOG immunization, the total numbers of parenchymal mononuclear cells and CD4 T cells (**A**) and the representative flow cytometric plots (**B**), the percentages (**C**), cell numbers (**D**), or geometric mean of fluorescence index (**E**) of cytokine-producing CD4 subsets were measured in the CNS lesions of EAE mice in the absence (filled circle) or presence (filled triangle) of HBOT, respectively. Those data of IFN-γ^+^ (Th1), IL-17A^+^ (Th17), and IFN-γ^+^/IL-17A^+^ (ex-Th17) CD4 subsets were shown in panel A, B, and C, respectively. All data are representative of two independent experiments and were presented as mean ± SEM from six mice in each group. The two-way ANOVA test was used for the statistical analysis. * < 0.05, **< 0.001, *** < 0.0001. (**F**) The IFN-γ and IL-17A productions of splenic T cells in culture supernatants were measured in the presence of TCR stimulations. The single-cell suspensions were isolated from the spleen of EAE mice with (open bar) or without (closed bar) HBOT on day 14 after MOG immunization. The splenic single-cell suspensions were counted then seeded as 2 × 10^6^ per well of 12-well plate. Subsequently, those cell suspensions were stimulated by TCR signaling in the presence of coating anti-CD3ε mAb of 0, 0.3, or 1 μg per well for a total culture of 48 h, respectively. Accordingly, culture supernatants were harvested and immediately stored at −80 °C refrigerators temporarily. Those cytokine concentrations of IFN-γ and IL-17A in culture supernatants were measured by the ELISA assay kits, respectively. All data are representative of three independent experiments and were presented as mean ± SEM. The two-way ANOVA test was used for the statistical analysis. * < 0.05, **< 0.001.

To investigate whether HBOT affects the inflammatory responses of autoreactive T cells, by flow cytometric analysis, we examined the inflammatory cytokines-producing CD4 T cells in the CNS from EAE mice treated with HBOT. The total numbers of parenchymal mononuclear cells (CD45^hi^) and CD4 T cell subset (CD45^hi^/CD3ε^+^/CD4^+^) were measured in the CNS lesions of EAE mice. Our results indicated that both total numbers of mononuclear cells and CD4 T cell subset were significantly diminished in the CNS lesions of HBOT-treated EAE mice as compared to vehicle control-treated mice, respectively (Figure 3A). As reported, Th1, Th17, and ex-Th17 are pathogenic CD4 T cell subsets in both the initiation and progression of autoimmune encephalomyelitis, respectively [1,28]. Accordingly, our results indicated that the percentages of IL-17A-producing CD4 T cells were significantly lower in the CNS of EAE mice receiving HBOT than those of vehicle control EAE mice with a normal room oxygen context (Figure 3B,C), even though the frequencies of Th1 and ex-Th17 cells were rarely distinguished between those two groups of EAE mice, HBOT-treated and vehicle control-treated mice, respectively (Figure 3C). Besides, the absolute cell numbers of those CD4 T cell subsets, including Th1, Th17, and ex-Th17 subsets, were further measured. Consequently, we found that the cell numbers of them were strikingly attenuated in the CNS lesions of HBOT-treated EAE mice as compared to those of vehicle control-manipulated EAE mice, respectively (Figure 3D). To further analyze the geometric mean of cytokine expression by the LSRII flow cytometer, we measured the mean fluorescence index (MFI) of cytokine in those T helper cells in the CNS lesions of EAE mice. We found that the MFI of IFN-γ and IL-17A were notably diminished in CNS-infiltrating Th1 and Th17 cells of EAE mice receiving HBOT as compared to those of vehicle control, respectively (Figure 3E). Furthermore, these results supported that HBOT alleviated the inflammatory responses of Th1, Th17, and ex-Th17 by downregulation of their cell numbers in the CNS lesions of EAE mice, partially among them, at least, significantly affecting the abundance of the Th17 subset and the productions of IFN-γ and IL-17A of T helper cells in the CNS lesions. To further determine whether HBOT modulates the inflammatory cytokine productions of T cells, on day 14 after MOG immunization, we respectively measured the secretions of IFN-γ and IL-17A of the splenic T cells, in the presence of TCR stimulation, of the HBOT-treated EAE mice. Our results demonstrated that the productions of IFN-γ of the splenic T cells were almost equal between the two EAE mice groups by each dose of anti-CD3ε mAb stimulation (Figure 3F, left panel), although being conversely different from the IFN-γ MFI of the encephalomyelitogenic Th1 cells in the CNS lesions. However, we investigated that the productions of IL-17A of the splenic T cells were markedly downregulated in HBOT-treated EAE mice as compared to those in vehicle control-treated EAE mice in the presence of TCR stimulation of each anti-CD3ε mAb dose (Figure 3F, right panel), consistent with the IL-17A MFI reduction in the pathogenic Th17 cells of the CNS lesions. Moreover, our findings suggested that HBOT modulated the IL17A production of the T cells in spleen and CNS lesion, at least to further promote its protective effects on the amelioration of autoimmune encephalomyelitis.

### 3.4. HBOT Diminishes the Productions of TNF-α and GM-CSF, and Boosts the Expressions of IL-4 and IL-10 of Encephalomyelitogenic CD4 T cell Subsets in the CNS Lesions

**Figure 4:**
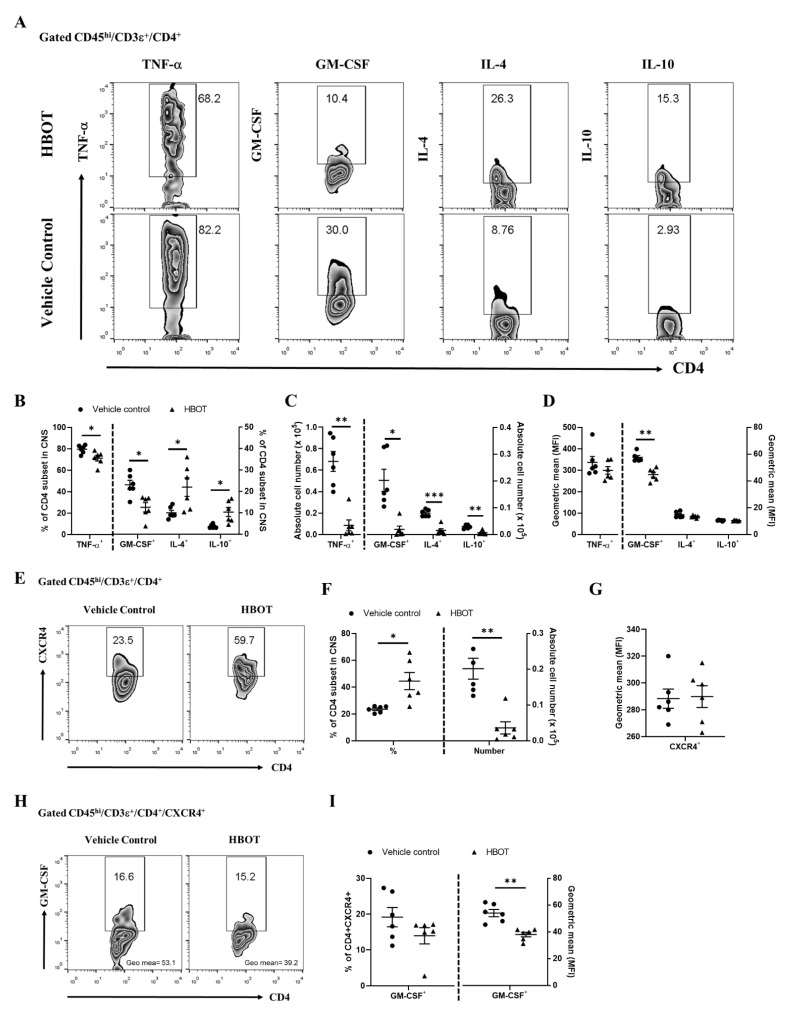
HBOT markedly attenuated the contexts of TNF-α and GM-CSF pro-inflammatory cytokines, as well as augmented the frequencies of IL-4 and IL-10 anti-inflammatory cytokines, in the CNS lesions-infiltrating CD4 T cell subsets. On day 14 after MOG_35–55_ immunization, the representative flow cytometric plots (**A**), the percentages (**B**), cell numbers (**C**), or geometric mean of fluorescence index (**D**) of cytokine-producing CD4 subsets were measured in the CNS lesions of EAE mice with (filled triangle) or without (filled circle) HBOT, respectively. The pro-inflammatory cytokines, TNF-α and GM-CSF, as well as anti-inflammatory cytokines, IL-4 and IL-10, were measured in the CD4 T cell subsets of the CNS lesions. All data are representative of two independent experiments and were presented as mean ± SEM from six mice in each group. The two-way ANOVA test was used for the statistical analysis. * < 0.05, **< 0.001. (**E**–**G**) The context of the CXCR4-expressing CD4 T cell subset was explored in the CNS lesions of EAE mice. On the day 14 after MOG immunization, the representative flow cytometric plots (**E**), the percentages ((**F**), left panel), absolute cell numbers ((**F**), right panel), and geometric mean fluorescence index (**G**) of CXCR4 in the CNS-infiltrating CD4 T cell subsets were measured in HBOT- or vehicle control-treated EAE mice. (**H**,**I**) The expression of GM-CSF in CXCR4-positive CD4 T cell subset. On the day 14 after MOG immunization, the representative flow cytometric plots (**H**), the frequencies ((**I**), left panel), or geometric mean of fluorescence index ((**I**), right panel) of GM-CSF in CD4 T cell subset were measured in the CNS lesions of HBOT-treated and vehicle control-treated EAE mice, respectively. All data are representative of two independent experiments were presented as mean ± SEM from six mice in each group. The two-way ANOVA test was used for the statistical analysis. * < 0.05, **< 0.001.

To investigate whether HBOT also modulates the pro-inflammatory responses of TNF-α and GM-CSF of the autoreactive T cell subsets in autoimmune encephalomyelitis [3], we further analyzed the percentages, cell numbers, and geometric mean of fluorescence index of those cytokines-producing CD4 T cell subsets (CD45^hi^/CD3ε^+^/CD4^+^) in the CNS lesions of EAE mice on day 14 after MOG immunization, respectively. Our results displayed that the percentages and cell numbers of TNF-α and GM-CSF in the CD4 T cell subsets of HBOT-treated EAE mice were significantly diminished as compared to those of vehicle control-treated EAE mice, respectively (Figure 4B,C, left panel). Besides, the geometric mean of fluorescence index of GM-CSF in the CNS-infiltrating CD4 T cell subsets was suppressed in the EAE mice receiving HBOT as compared to that in the vehicle control-treated EAE mice (Figure 4D, left panel). On the contrary, we found that the geometric mean of TNF-α expression was almost equal between those two EAE mice groups (Figure 4D, left panel). Therefore, these results suggested that HBOT profoundly weakened the pro-inflammatory responses mediated by the productions of TNF-α and GM-CSF of the autoreactive CD4 T cell subsets in the CNS lesions, respectively, somewhat in relation to its protective outcome on the progression of autoimmune encephalomyelitis. To further explore whether HBOT modulates the immunosuppressive cytokines in autoimmune encephalomyelitis [28], we additionally tested the productions of IL-4 and IL-10 in the CD4 T cell subsets of the CNS lesions from EAE mice on day 14 after MOG immunization. Our results showed that the frequencies of the IL-4- and IL-10-secreting CD4 T cell subsets were significantly boosted in the CNS lesions of HBOT-treated EAE mice as compared to those of vehicle control-treated EAE mice, respectively (Figure 4B, right panel). However, due to much lower numbers of CNS-infiltrating parenchymal mononuclear cells of the HBOT-treated mice, we analyzed that the absolute cell numbers of those CD4 T cell subsets were significantly much lower in the amount of EAE mice treated with HBOT than those of vehicle-treated mice (Figure 4C, right panel). Nevertheless, our data showed that the geometric means of IL-4 and IL-10 fluorescence index of the CNS-infiltrating CD4 T cell subsets were almost similar between those two EAE mice groups, respectively (Figure 4D, right panel). Consequently, these results suggested that HBOT respectively promoted the productions of IL-4 and IL-10 immunosuppressive cytokines of CD4 T cells in the CNS lesions, thus at least further contributing to the attenuation of the EAE clinical manifestations. As reported [26], CXCR4 is a potent chemokine receptor of encephalomyelitogenic T cells in the development of autoimmune encephalomyelitis. To further examine whether HBOT modulates the CXCR4 expression of autoreactive T cells in autoimmune encephalomyelitis, we measured the percentage, cell number, and geometric mean of fluorescence index of the CXCR4-expressing CD4 T cell subsets (CD45^hi^/CD3ε^+^/CD4^+^/CXCR4^+^) in the CNS lesions of EAE mice on day 14, respectively. Accordingly, our data showed that the frequency of CXCR4-expressing CD4 T cells was significantly boosted in the CNS lesions of EAE mice treated with HBOT as compared to vehicle control-treated mice (Figure 4F, left panel), even though we found that the cell number of CXCR4-expressing encephalomyelitogenic CD4 T cells in the CNS lesions was much lower in the EAE mice with HBOT treatment than that in mice without treatment (Figure 4F, right panel). Besides, the geometric mean of the CXCR4 fluorescence index in the autoreactive CD4 T cells was considerably similar between the two EAE mice groups (Figure 4G). Moreover, these results suggested that HBOT augmented the frequency of CXCR4 in the CD4 T cell subset of the CNS lesions from the treated EAE mice, even if this was inconsistent with the protective outcome of EAE after the treatment of HBOT, somewhat indicating a dispensable effect of those CXCR4-expressing CD4 T cells. To further measure the GM-CSF production in the CXCR4-positive CD4 T cell subset (CD45^hi^/CD3ε^+^/CD4^+^/CXCR4^+^), the frequencies of GM-CSF-producing in these CD4 T cell subsets were equal between HBOT-treated and vehicle control-treated EAE mice. Nevertheless, we found that the geometric mean of fluorescence index of GM-CSF in those CD4 T cell subsets was significantly downregulated in the CNS lesions from EAE mice with HBOT as compared to vehicle control. These results suggested that HBOT markedly attenuated the pathogenicity of the CXCR4-expressing encephalomyelitogenic CD4 T cells via the downregulation of GM-CSF.

## 4. Discussion

Our results demonstrated that HBOT alleviated the clinical severity and progression of MOG-immunized EAE. In this study, we used a semi-therapeutic manner to treat those EAE mice prior to the effector phase of the disease, meaning that the HBOT treatment was administrated at an earlier stage of the initiation phase immediately before the disease onset. We treated EAE mice with HBOT from day 8 after MOG immunization and the average disease onset in our laboratory was 10.4 days. Rationally, we suggested that it is necessary for effective HBOT treatment to manipulate it at an earlier time point nearby disease onset, either before or a little time after onset. Additionally, our evidence partially adds a scientific basis to those studies investigating the protective roles of HBOT in MS patients or neuronal cells [7,8,9]. In 1983, Fischer and colleagues enrolled MS patients for the HBOT treatment trial, and they found a positive initial response for MS patients [7]. Nevertheless, they still did not prefer to recommend HBOT treatment for MS unless a further confirmation of a long-term follow-up. Recently, HBOT was applied for MS patients and supported a protective role of HBOT in the symptoms of disease [8,9]. The underlying mechanisms were proposed to be related to adhesion molecule ICAM-1 of endothelial cells, maintaining blood brain barrier (BBB) permeability, or the ER stress homeostasis in neuronal cells, eventually, in part, contributing to the protective role of HBOT mentioned as above. However, several previous clinical trials before 2010 indicated that HBOT was not recommended for the treatment of MS [14,15,16,17,18]. Those trials enrolled MS patients who received HBOT treatment as an adjunct to therapy. The overall therapeutic benefits of HBOT-treated MS patients did not be markedly different from those of placebo control patients. These trials were individualized to use different hyperbaric oxygen pressure, treatment frequency, and treatment time. Besides, placebo controls were defined differentially among those trials and the evaluations of clinical symptoms were not equally recorded as well as analyzed according to similar standard rules. Most importantly, in those clinical trials, MS patients received HBOT at a later stage of disease while their clinical manifestations had worsened and HBOT was only as an adjunct to therapy after other drug medications. On the contrary, our results emphasized that HBOT ameliorated the EAE disease while mice received HBOT in a much earlier stage prior to the effector phase of the disease, further partially explaining why those MS patients with a later stage in previous clinical trials did not benefit from HBOT [18]. We administrated two rounds of five consecutive HBOT treatments in EAE mice, but T cell context in the CNS lesions was analyzed on day 14 after MOG immunization. Inclusion of the second time point of analysis would be more comprehensive to understand the effectiveness of HBOT in EAE. Nevertheless, because after or during the second round of HBOT, the clinical scores of EAE mice still continued to increase, but not completely reversed, we expected that HBOT in the current design may still confer certain levels of protection by skewing the T cell subset deviation toward the immunomodulatory context at the earlier stage of the effector phase, thus it may also promote the amelioration of EAE in the later stage of the disease. Furthermore, in-depth studies are certainly required. Other reports also supported our results in spinal cord injury and traumatic brain injury, where the interventional time of animals when they received HBOT at an earlier stage critically determined the therapeutic efficacy [29,30].

In this study, HBOT treatment promoted the anti-inflammation response as well as alleviated the disease severity of EAE by the downregulation of parenchymal mononuclear infiltration. In addition, we found that HBOT dampened the inflammatory cytokines and augmented the immunosuppressive cytokines of CD4 T cells from CNS lesions of EAE mice. Our findings were somewhat supported by the previous reports. Recently, HBOT is usually employed to treat neurological disorders, manifested by anti-inflammatory effects against disease progression. One of the HBOT underlying mechanisms is attributed to the modulations of cytokines, promoting immunosuppressive cytokine as well as retarding the pro-inflammatory cytokines. In experimental spinal cord injury, HBOT treatment increased the production of IL-10 and VEGF as well as attenuated the overproduction of IL-1β η and TNF-α [11]. HBOT attenuated the TNF-α production but not IL-1β to alleviate the neuropathic pain caused by chronic constrictive injury [31]. In the traumatic brain injury model, HBOT augmented the production of immunosuppressive IL-10 to promote the effects of anti-inflammation [32,33]. HBOT increased the production of IL-4 and IL-13 to exert the anti-inflammatory effects in spinal cord injury and neurodegenerative diseases, respectively [34,35]. HBOT significantly shifted from M1-like to M2-like macrophages to further promote the amelioration of spinal cord injury [34]. HBOT attenuated the inflammasome resulting in the downregulation of IL-1β as well as IL-18, and its related signaling pathways [30,36,37], suggesting an inhibitory effect of HBOT on the inflammation context. Consistent with those previous reports, our findings additionally provided a scientific basis to support that HBOT attenuated the IL-17A, TNF-α, and GM-CSF productions of CD4 T cells from CNS lesions of EAE mice. However, HBOT boosted the expression of IL-4 and IL-10 of CD4 T cells from CNS lesions. Taken together, HBOT has a protective role to mediate anti-inflammatory effects on tissue and T cells, eventually somewhat contributing to the amelioration of diseases. On the other hand, a protective role of HBOT is attributed to maintain the BBB integrity, resulting in the downregulation of mononuclear leukocyte infiltration into CNS. In the traumatic brain injury, HBOT has been evidenced to reduce BBB disruption and decrease its permeability [33,35,38]. In MS, HBOT significantly downregulated the expression of ICAM-1 of brain endothelial cells, implicating an inhibition of inflammatory cell migration across the blood brain barrier (BBB) [9]. ICAM-1 is already investigated to regulate the CNS entry of autoreactive Th1 and Th17 cells of EAE disease in an earlier stage [39]. Moreover, Th1 and Th17 cells respectively produce IFN-γ and IL-17A to critically disrupt the BBB in an earlier stage of MS and EAE [40,41,42]. Similarly, GM-CSF breaks down the BBB and promotes the migration of monocytes across the BBB into CNS [43]. In line with these previous reports, we found that the absolute cell numbers of mononuclear leukocytes were indeed much lower in HBOT-treated EAE mice than those of vehicle-treated EAE mice, in part, supporting an inhibitory role of HBOT in the migration of mononuclear leukocytes into CNS. Besides, we found that HBOT downregulated Th17 cells and GM-CSF-producing CD4 T cells in the CNS lesions of EAE. It is likely to explain that HBOT dampened the Th17 cells and GM-CSF production of CD4 T cells and then promoted the integrity of BBB, subsequently preventing from CNS entry of leukocytes. Moreover, a protective role of HBOT in neurological disorders is also attributed to change the oxygen context of CNS lesions. HBOT is evidenced to suppress the HIF-1α expression by the increase of oxygen context in CNS lesions of neurological disorders and to promote the angiogenesis via the production of VEGF [12,44,45]. HIF-1α was induced by a hypoxia condition to regulate the development of Th17 cells during the pathogenesis of EAE [46]. Consistent with our results, HBOT attenuated the Th17 cell responses in the CNS lesions of EAE.

T helper cell subsets are pathogenic in the development of MS and EAE. In this study, we found that HBOT affects the frequency of Th17, but not Th1 as well as ex-Th17 cells. In line with previous studies [21], Th17 cell is the main encephalitogenic T population in EAE [47] and its cytokine releasing IL-17A is dispensable in the development of neuroinflammation in autoimmune encephalomyelitis [48]. Consequently, we suggested that HBOT-mediated attenuation of EAE, at least, is partially correlated to the reduction of Th17 differentiation in the CNS lesions. Besides, our data also indicated that GM-CSF- and TNF-α-producing CD4 T cell subsets were suppressed in HBOT-treated EAE mice. Consistent with previous studies, GM-CSF-deficiency mice is resistant to EAE induction [49]. Adoptive transfer of GM-CSF-deficient T cells but not IFN-γ- or IL-17A-deficient T cells, failed to induce EAE disease [25,26]. A novel CD4 T helper cell subset, which expressed GM-CSF and CXCR4, was already defined in MS and played an encephalitogenic role in disease progression [26]. On the other hand, HBOT was evidenced to promote SDF-1/CXCR4 in spinal cord injury [29]. Unexpectedly, we found that the frequency of CXCR4 in the CD4 T cell subset was upregulated from the CNS lesions of HBOT-treated EAE mice. Nevertheless, due to GM-CSF downregulation in CNS-infiltrating CD4 T cell subset, we summarized that the higher CXCR4 expression of CD4 T cells slightly affects the EAE disease after HBOT treatment, suggesting the GM-CSF as a critical secreting weapon. In addition, TNFα synergizes with both IL-17A and GM-CSF to promote the migrations of neutrophils and monocytes [44,50], and its deficiency attenuates the EAE in mice. These previous reports further supported our results that the inflammatory context significantly downregulated in the CNS lesions of HBOT-treated EAE mice, providing a scientific basis to elucidate a protective effect of HBOT in EAE.

## 5. Conclusions

In this study, we demonstrated that semi-therapeutic HBOT alleviated the clinical manifestations and progression of MOG-immunized EAE in which mice received treatment in an earlier stage prior to the effector phase of disease. HBOT significantly dampened the frequency of Th17 cells, but not Th1 and ex-Th17, in the CNS lesions of EAE mice, even though we found that the absolute cell numbers of those T cell subsets were markedly decreased in HBOT-treated EAE mice. Both the geometric mean of IFN-g and IL-17A expression were downregulated in CNS-infiltrating Th1 and Th17 cells of EAE mice in the presence of HBOT, respectively. Moreover, we found that TNFα and GM-CSF production were strikingly retarded in CD4 T cell subset in the CNS lesions of HBOT-treated EAE mice. Besides, the immunosuppressive IL-4 and IL-10 were profoundly boosted in CD4 T cell subsets in the CNS lesions of EAE mice with HBOT. Conclusively, our results indicated that semi-therapeutic HBOT is effective for the treatment of MOG-immunized EAE via the modulation of T cell subsets.

## Figures and Tables

**Table 1:** Clinical manifestations of HBOT-treated EAE mice.

Treatment Group	EAE Incidence	Day(s) of Onset (Mean ± SEM)	Maximal Clinical Score (Mean ± SEM)	Peak Phase with Maximal Clinical Score (Mean ± SEM)
Vehicle control	14/14 (100%)	10.6 ± 0.5	3.1 ± 0.1	3.2 ± 0.8
HBOT	12/14 (86%)	12.3 ± 1.0	2.2 ± 0.3	2.4 ± 0.4
*p* value	NA	0.1439, ns	0.0095 *	0.7694, ns

The unpaired Student *t*-test was used for statistical analysis. * < 0.05. NA: non-available.
